# Vibrant symbiosis: Achieving reciprocal science outreach through biological art

**DOI:** 10.1371/journal.pbio.3000061

**Published:** 2018-11-30

**Authors:** Lindsey E. Lopes, Sarah J. Waldis, Stephanie M. Terrell, Kristin A. Lindgren, Louise K. Charkoudian

**Affiliations:** 1 Department of Biology, Haverford College, Haverford, Pennsylvania, United States of America; 2 Department of Chemistry, Haverford College, Haverford, Pennsylvania, United States of America; 3 Health Studies Program, Haverford College, Haverford, Pennsylvania, United States of America

## Abstract

Scientific outreach efforts traditionally involve formally trained scientists teaching the general public about the methods, significance, and excitement of science. We recently experimented with an alternative “symbiotic outreach” model that prioritizes building a reciprocal relationship between formally trained and “outsider” scientists to facilitate active two-way communication. Herein, we present the results of our outreach effort involving college students and adults with intellectual and developmental disabilities working together to make biological and multimedia art. By discussing the steps others can take to cultivate reciprocal outreach within their local communities, we hope to lower the barrier for widespread adoption of similar approaches and ultimately to decrease the gap between formally trained scientists and the general public.

## Abstract 2

This Community Page article presents an alternative outreach model that prioritizes building a reciprocal relationship between scientists and outsiders, describes an example involving college students and adult artists with intellectual and developmental disabilities working together to create BioArt, and discusses how others can cultivate similar opportunities within their local communities.

## Introduction

The persistent and growing inequity between the knowledge and resources of formally trained scientists and the general public makes it difficult for the public to relate to the work of scientists and even to distinguish pseudoscience from the real thing [1]. This is especially true for groups traditionally excluded from science education and the scientific workforce, such as people with disabilities [2]. Science outreach presents a promising means of bridging this widening gap by cultivating fertile ground for formally trained scientists and “outsider scientists” to interact. Drawing on the term “outsider artist,” which describes a working artist without formal training [3], here, we use “outsider scientist” to refer broadly to anyone engaging in science without formal training. The idea of an “outsider scientist” recognizes the scientific curiosity and potential within each of us, regardless of professional background, while also purposefully calling attention to the problematic, sometimes exclusive nature of science as a field.

While traditional science outreach efforts have often focused on a one-way transfer of information from “experts” to “learners,” we recently experimented with an alternative outreach model that prioritizes building a reciprocal relationship between formally trained and outsider scientists. By explicitly valuing the backgrounds and goals of both groups, this model draws on active two-way communication as a more productive way to learn and retain information [4], while also creating an opportunity to enhance the mutual appreciation of traditionally disparate disciplines and people.

We experimented with this model of reciprocal outreach through “Symbiosis: Art, Science, & Community,” a collaboration between college students and adults with intellectual and developmental disabilities (IDD). The many expected and unexpected benefits of this collaboration inspired us to share our story with the larger scientific community to encourage others to find creative ways to engage with the public. Additionally, we aim to reduce the barrier to widespread adoption by providing specific and feasible guidelines for scientists to develop reciprocal partnerships of their own.

## Symbiosis: A reciprocal partnership between college students and adults with intellectual disabilities

Symbiosis was initiated by two Haverford College undergraduate students (LEL and SJW) interested in both biology and disability studies, who wanted to utilize science outreach as a means to bring together two groups that do not often have the opportunity to interact: college students and adults with IDD. Following a desire to explore creative ways of viewing and communicating science, they initiated a partnership with a local vocational art studio for adults with IDD, the Center for Creative Works (CCW) in neighboring Wynnewood, Pennsylvania. The CCW artists expressed interest in engaging with the local community, exploring the concept of organisms you cannot see (like germs), and sharing their considerable technical knowledge of artistic practices with college students.

Students in self-contained special education classes, which often includes students with IDD, frequently have minimal exposure to science despite science education being important for developing critical thinking and problem-solving skills [2]. In fact, the majority of the CCW artists had never been in a science laboratory prior to beginning the project. The project therefore was designed to draw on the interests and expertise of both groups, and the partnership was grounded in the principle of “symbiosis.” A term technically defined as a mutually beneficial relationship between organisms (think Nemo and the sea anemone), symbiosis for us served as a more poetic statement that everyone involved had both something to teach and something to learn.

The two college students collaborated with a faculty member (KAL) to integrate Symbiosis into an interdisciplinary disability studies course and with a faculty member in chemistry (LKC) to design ways to paint with vibrantly pigmented *Streptomyces* bacteria to create “BioArt.” Many scientists study the pharmacologically relevant molecules produced by *Streptomyces* (such as antibiotics); these strains can also be repurposed as a source of biopigments because the polyaromatic polyketides produced by the organism imbue the strains with their characteristic hues. Meanwhile, the CCW artists identified three different realms of art making—woodworking, mixed-media sculpture, and fiber arts—to teach the college students. They then collaborated on pieces inspired by the time in the lab. Thus, *Streptomyces* catalyzed our discussions of the connections between art and science and served as a vehicle for us to share our unique areas of expertise.

**Fig 1:**
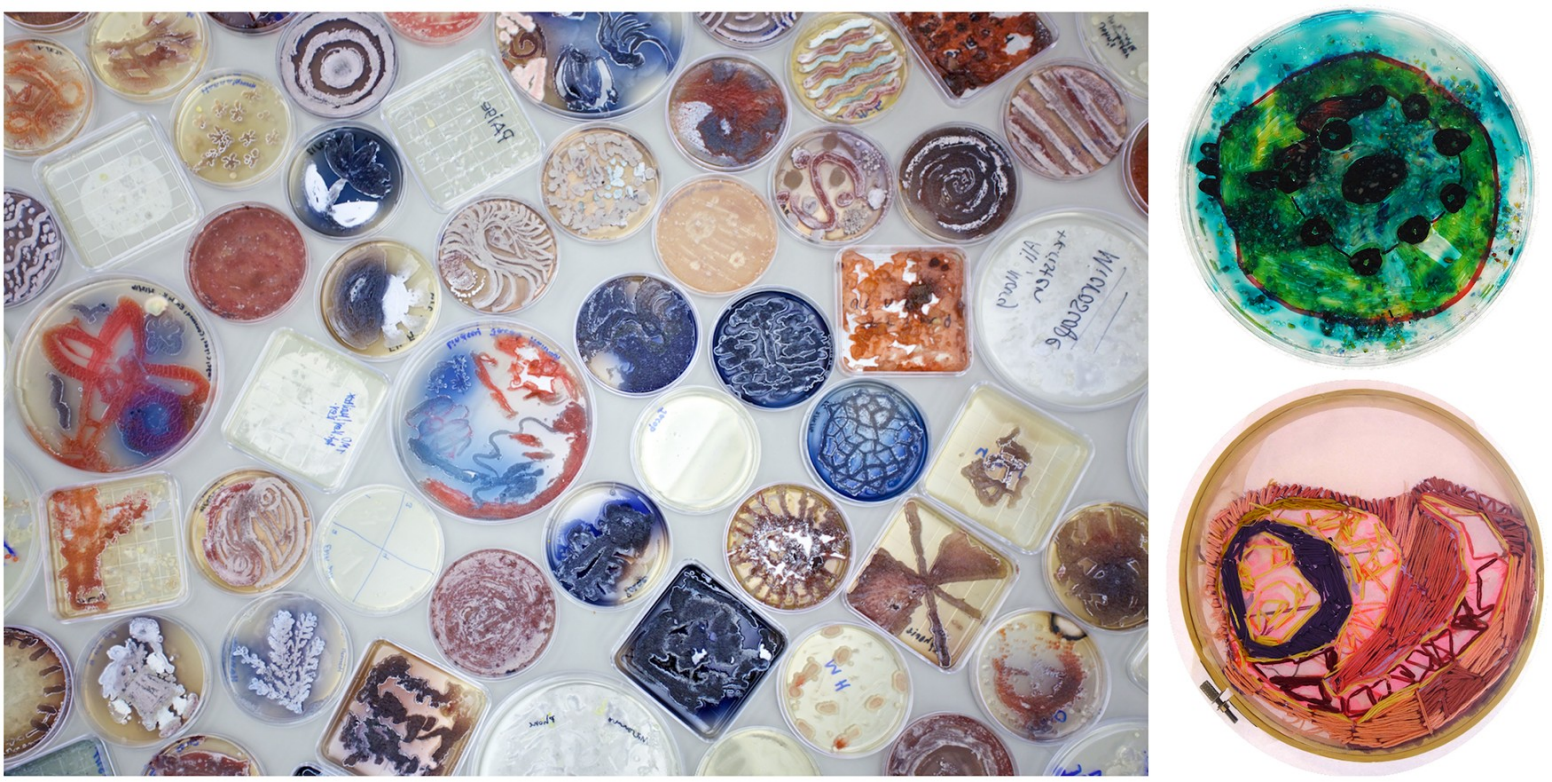
Photographs of BioArt (left) and biology-inspired multimedia art (right) created through a symbiotic outreach project between 20 adult artists with intellectual and developmental disabilities and Haverford College undergraduate students. *Photos by Caleb Eckert*.

Over the course of three weeks, the students enrolled in the disability studies course, and a group of artists from CCW met in a laboratory space at Haverford College to explore making BioArt as a means to exchange knowledge. Detailed protocols for how *Streptomyces* can be utilized in BioArt were pulled from *In Living Color*: *Bacterial Pigments as an Untapped Resource in the Classroom* [5]. Using paint brushes and sterile cotton swabs, students (with and without prior laboratory experience) and CCW artists streaked a variety of *Streptomyces* strains onto petri dishes filled with nutrient-rich agar, creating living artwork that remained invisible until the cultured bacteria grew to produce a stunning array of colors, textures, and patterns (Fig 1, left). After observing and documenting their BioArt by eye and with a microscope, the college students travelled to the CCW studio to learn techniques such as needlepoint and work with materials such as Mod Podge. Working side-by-side with the artists, they made pieces that reflected their shared experience of working with bacteria and observing it grow (Fig 1, right). Splitting time between the lab and the studio allowed the college students to achieve their goal of finding creative ways to communicate science and allowed the artists to achieve their goal of finding creative ways to share their craft.

Students and artists later shared their work through a public exhibition entitled “Symbiosis: Art, Science, & Community” and an exhibition catalog with essays about the partnership, as well as photographs of the BioArt, the mixed-media art work, and the students and artists working together in the laboratory and art studio. The project was recreated by another Haverford College undergraduate student enrolled in the disabilities studies course (SMT) in a subsequent year, expanding the impact of this project to more students and artists.

Scientific outreach is traditionally rooted in “top-down” goals: it gives scientists a chance to show the public what they do and provides an opportunity to improve the scientific literacy of the public through hands-on experience [6,7]. In line with these goals, artists that completed the BioArt project communicated a newfound interest in working with bacteria as well as a sense of comfort in the notoriously exclusionary laboratory environment. These reactions echoed what we have heard previously when creating BioArt with high school students from backgrounds under-represented in the sciences.

The overriding aim of our outreach model, however, was to move beyond a one-way teaching experience and to design a project that incorporated the expertise of both the formally trained and outsider scientists. In the Symbiosis exhibition catalogue, Samantha Mitchell, Arts and Exhibition Coordinator at CCW, writes:

Haverford College is a ten-minute drive from CCW, yet very few of our participants—some of whom have worked at this location for over 30 years—have ever set foot on the campus before beginning the project. Having the opportunity not only to visit a college campus but to belong there, to have a purpose and place within the community, is invaluable for us. Equally as meaningful is the experience that the Haverford students are invited to have with our artists. Engaging with the ID [intellectual disability] community in a prolonged way allows the opportunity for barriers and stigma to dissolve, and working together on a creative project heightens the possibility for real connection. With a project like SYMBIOSIS, we hope to make lasting changes in the way people view the ID community: as peers, potential collaborators, and uniquely creative people.

Early discussions with CCW about what they wanted to gain from a collaboration made possible the kind of meaningful interaction that Mitchell describes. Explicitly asking what our partners wanted to gain from a collaboration with us helped to avoid the top—down assumption that we knew what was best for them and allowed us to imagine what our outreach could achieve beyond just bacteria. Furthermore, it gave us what we, as formally trained scientists, dream of when entering this career: the ability to utilize science as a tool to enact change in the world that extends far outside of our lab space.

Moving toward a model of symbiotic science outreach means also reflecting on how the formally trained scientists benefited from engaging with the public. During the process of designing and implementing the BioArt experience, the college students reported anticipated benefits (e.g., improved ability to communicate science in creative ways) and many unexpected benefits. For example, during our time in the lab, the artists continuously “disrupted” typical lab rules. They punctured the agar, did not always sterilize tools prior to streaking or changing strains of bacteria, and asked if the agar was edible, making the overall process less goal-oriented and ironically more experimental than we, as budding scientists, had planned. We worried that these deviations from the boundaries of the lab space we had come to internalize would ruin the experiment, but after seeing the breathtaking results, we began to understand making BioArt as a creative scientific process rather than two distinct creative and scientific processes jammed together. We were struck by the creativity inherent in the scientific method, and we were reminded of how scientists frequently adapt protocols in the face of unexpected challenges and reframe conclusions based on surprising results. The artists pushed us to explore multiple ways to grow bacteria, a practice called “divergent thinking” that has been shown in science and engineering pedagogy to improve the creative thinking skills of students [8]. Indeed, as Janet Stemwedel wrote, “we find out the difference between objective facts and subjective impressions of the world by actually sharing a world with other people whose subjective impressions about the world differ from our own” [9]. Scientists are trained to value a certain set of diverse perspectives within their field (peers, colleagues, reviewers, etc.), so why not see what happens when we open that up even further?

## What steps can you take to cultivate symbiotic science outreach?

**Fig 2:**
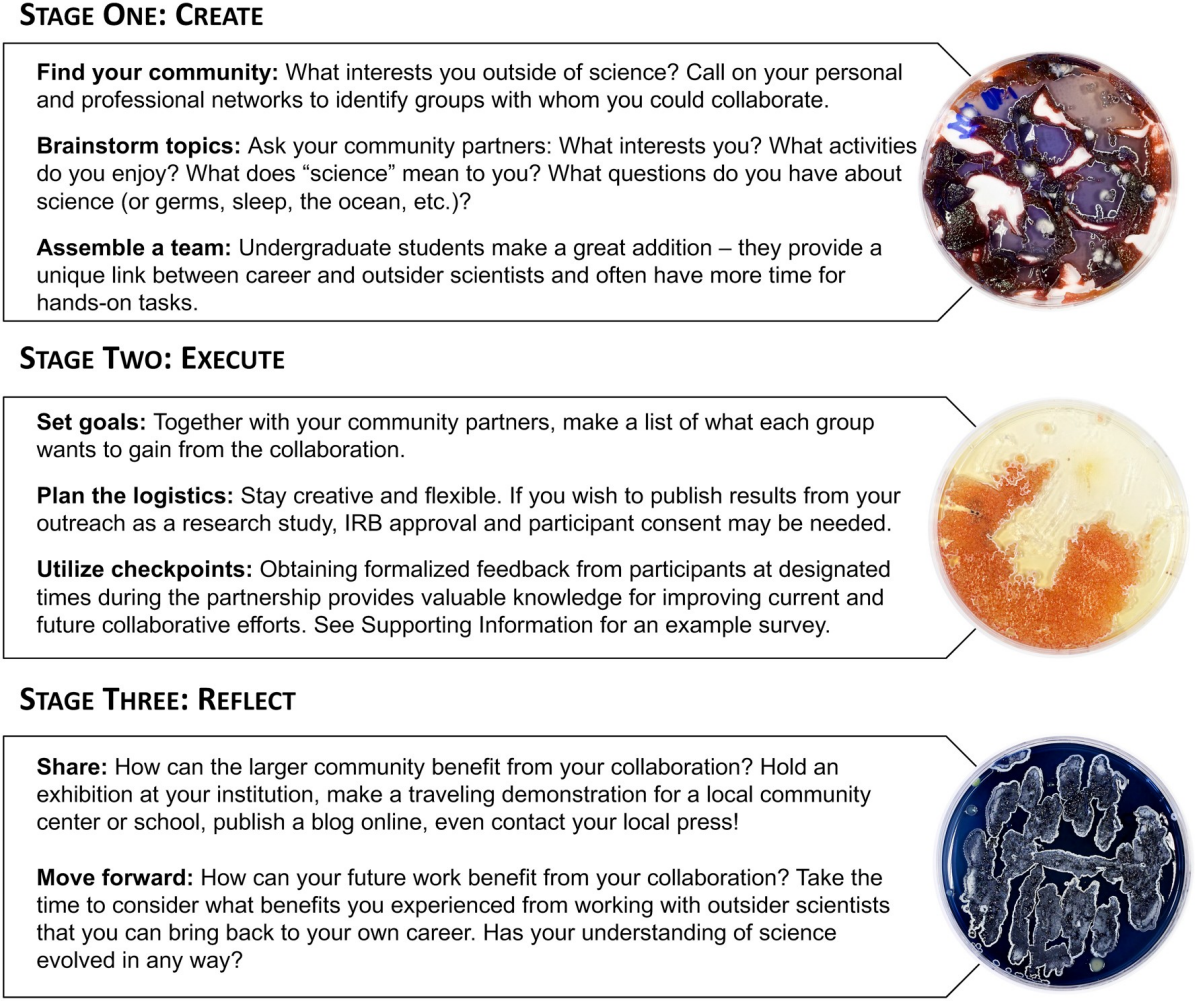
Tips for symbiotic science outreach. *Photos by Caleb Eckert*.

**Fig 3:**
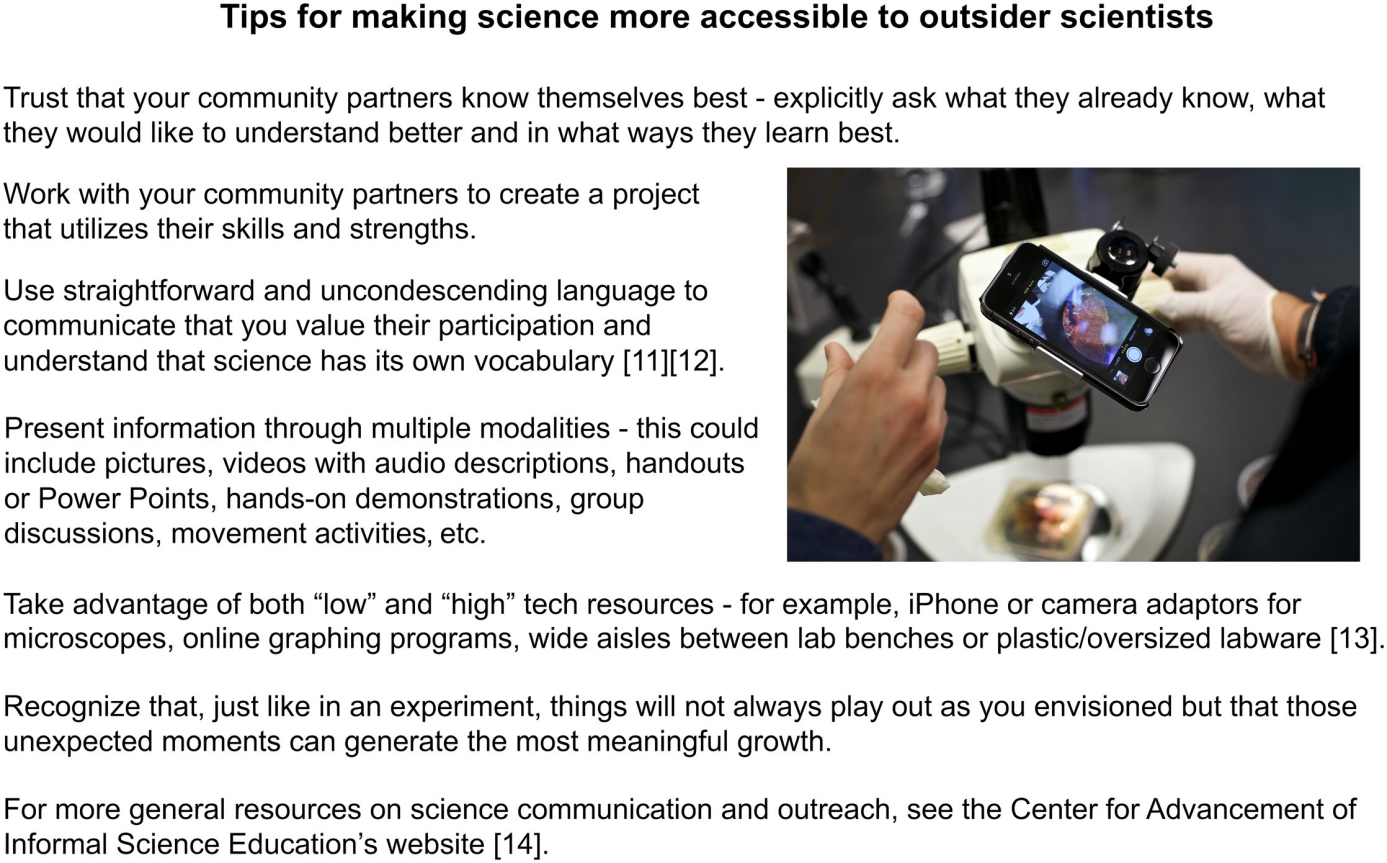
How can we make science more accessible? *Photos by Caleb Eckert*.

Above all, carrying out symbiotic science outreach requires a simple yet, in many ways, novel commitment to value the curiosities of each person involved, regardless of their technical knowledge of science. We hope the previous section has convinced you that many can benefit from this kind of approach, yet we also know from experience that this framework can feel easier said than done. We created Fig 2 to illustrate practical suggestions for how to transform a general science outreach project workflow into one that emphasizes cultivating a reciprocal relationship between scientists and outsiders. Fig 3 provides a guide to making science more universally accessible to outsider scientists, including people with disabilities [10–14].

## Conclusion

With the ongoing push for scientists to bridge the gap between the “ivory tower” and the general public, outreach projects are a particularly appealing way of engaging with outsiders. However, many scientific outreach projects are focused solely on improving the knowledge and scientific literacy of the general public. Here, we advocate for the importance of collaborative relationships and urge other scientists to think creatively about how to design symbiotic scientific outreach projects. When we began to think about a project that could bring together our interests in biology and disability studies, it was hard to picture what that type of outreach would look like. We hope that our story and framework illustrates the feasibility and importance of creating reciprocal outreach partnerships that benefit formally trained and outsider scientists alike.

## Abbreviations

CCW: Center for Creative Works
IDD: intellectual and developmental disabilities
